# Isolation, purification and PEG-mediated transient expression of mesophyll protoplasts in *Camellia oleifera*

**DOI:** 10.1186/s13007-022-00972-1

**Published:** 2022-12-22

**Authors:** Sufang Li, Rui Zhao, Tianwen Ye, Rui Guan, Linjie Xu, Xiaoling Ma, Jiaxi Zhang, Shixin Xiao, Deyi Yuan

**Affiliations:** 1 Key Laboratory of Cultivation and Protection for Non-Wood Forest Trees, Ministry of Education, Central South University of Forestry and Technology, Changsha, 410004 Hunan China; 2 Key Laboratory of Non-wood Forest Products of State Forestry Administration, Central South University of Forestry and Technology, Changsha, 410004 Hunan China; 3 Department of Plant Breeding, Swedish University of Agricultural Sciences, Alnarp, 23053 Skåne, Sweden

**Keywords:** *Camellia oleifera*, Leaf mesophyll, Protoplast isolation, Purification, PEG, Transient transformation

## Abstract

**Background:**

*Camellia oleifera* (*C. oleifera*) is a woody edible oil crop of great economic importance. Because of the lack of modern biotechnology research, *C. oleifera* faces huge challenges in both breeding and basic research. The protoplast and transient transformation system plays an important role in biological breeding, plant regeneration and somatic cell fusion. The objective of this present study was to develop a highly efficient protocol for isolating and purifying mesophyll protoplasts and transient transformation of *C. oleifera*. Several critical factors for mesophyll protoplast isolation from *C. oleifera*, including starting material (leaf age), pretreatment, enzymatic treatment (type of enzyme, concentration and digestion time), osmotic pressure and purification were optimized. Then the factors affecting the transient transformation rate of mesophyll protoplasts such as PEG molecular weights, PEG4000 concentration, plasmid concentration and incubation time were explored.

**Results:**

The in vitro grown seedlings of *C. oleifera* ‘Huashuo’ were treated in the dark for 24 h, then the 1st to 2nd true leaves were picked and vacuumed at − 0.07 MPa for 20 min. The maximum yield (3.5 × 10^7^/g·FW) and viability (90.9%) of protoplast were reached when the 1st to 2nd true leaves were digested in the enzymatic solution containing1.5% (w/v) Cellulase R-10, 0.5% (w/v) Macerozyme R-10 and 0.25% (w/v) Snailase and 0.4 M mannitol for 10 h. Moreover, the protoplast isolation method was also applicable to the other two cultivars, the protoplast yield for ‘TXP14’ and ‘DP47’ was 1.1 × 10^7^/g·FW and 2.6 × 10^7^/g·FW, the protoplast viability for ‘TXP14’ and ‘DP47’ was 90.0% and 88.2%. The purification effect was the best when using W buffer as a cleaning agent by centrifugal precipitation. The maximum transfection efficiency (70.6%) was obtained with the incubation of the protoplasts with 15 µg plasmid and 40% PEG4000 for 20 min.

**Conclusion:**

In summary, a simple and efficient system for isolation and transient transformation of *C. oleifera* mesophyll protoplast is proposed, which is of great significance in various aspects of *C. oleifera* research, including the study of somatic cell fusion, genome editing, protein function, signal transduction, transcriptional regulation and multi-omics analyses.

**Supplementary Information:**

The online version contains supplementary material available at 10.1186/s13007-022-00972-1.

## Introduction

*Camellia oleifera* (*C. oleifera*) is a valuable oilseed crop belonging to the genus *Camellia* of the Theaceae family and is mainly distributed in many provinces in southern China and Southeast Asian countries such as Vietnam, India and Japan [1, 2]. Camellia seed oil, rich in unsaturated fatty acids, vitamins, minerals, and other bioactive compounds, is used extensively in China as high-quality edible oil and reputed as ‘Oriental Olive Oil’ [3]. Moreover, camellia seed oil not only can effectively prevent the development of cardiovascular diseases, but also has anti-inflammatory and antioxidant capabilities [4]. Hence, camellia seed oil has become increasingly popular. However, the market still lacks improved *C. oleifera* varieties due to the obstacles in conventional breeding. As a cross-pollinated plant, *C. oleifera* possesses a highly heterozygous state in the genetic background. Conventional breeding in *C. oleifera* bears a long breeding cycle and offspring with complex and genetically unstable trait. The application of modern biotechnology can help solve these problems. Somatic cell fusion technology breaks the barriers of hybridization between species in biology, enables two species that cannot be sexually hybridized to perform asexual hybridization, and creates new varieties with excellent traits of both species through screening and purification [5]. The protoplast culture and fusion technique enlighten the genetic improvement for new varieties of *C. oleifera*. The prerequisite for utilizing this technique is to obtain a large quantity of highly viable protoplasts.

Plant protoplasts were the living material of plant cells by removing the cell wall and including the protoplasm and plasma membrane [6]. Due to the absence of cell walls, plant protoplasts have been widely used for genetic transformation, cell fusion, and somatic mutation [7–10]. In addition, plant protoplasts have totipotency and can regenerate into new similar individuals under appropriate conditions [11]. Somatic hybridization with viable protoplasts can break the reproductive barriers in the process of sexual hybridization or distant hybridization, and create new germplasm or new varieties that cannot be obtained by conventional breeding [12]. At present, cell fusion technology has been successfully applied in citrus [12, 13], cotton [14], oilseed rape [15] and other plants.

In previous studies, protocols for protoplast isolation have been very successful in herbaceous plants such as wheat [6], maize [16], rice [17], carrot [10], *Arabidopsis* [18], and perennial ryegrass [19]. Nevertheless, in woody plants the development of protoplast isolation technology has only been reported in citrus [20, 21], apricot [22], peach [23], tea plants [24, 25], *Populus* [26] and *Robinia pseudoacacia* L. [27].

Protoplasts could be isolated from different plant organs by enzymatic digestion [28]. Many factors including the enzyme type and concentration, osmotic pressure, enzyme digestion time and purification method could affect the enzymatic digestion efficiency [29, 30]. Most research has shown that the isolation conditions for protoplasts vary greatly among different tissues of the same species [31, 32]. For example, the enzyme solution ratio and duration of enzyme application, were different among *Robinia pseudoacacia* L. mesophyll and callus [27]. Therefore, it is generally necessary to evaluate a protoplast isolation system separately of different organs in the same plant.

In recent years, due to the advantages of rapidity and high efficiency, the plant protoplast transient expression system has been widely used in all types of research such as subcellular localization of proteins, molecular interaction, and signal transduction [33–35]. There are several commonly used transient transformation methods, such as the polyethylene glycol (PEG)-mediated one. Due to the high transformation efficiency of PEG-mediated method, it is widely applied in molecular and cellular studies in plants [36, 37]. The protoplast transient expression system plays an increasingly important role in genomics and proteomics research [38]. At present, protoplast transient expression systems have been established for many plant species, such as *Arabidopsis* [39], rice [40], barley [41], grapevine [42], poplar [26], and tea plants [25], and are widely used in basic research. To date, there are no reports of transient expression system using mesophyll-derived protoplasts in *C. oleifera*. A rapid and convenient protoplast transient transformation technique would be particularly useful for testing gene function or exploring some new technologies, such as genome editing in *C. oleifera*.

Protocols for the isolation and purification of protoplasts from *C. oleifera* suspension have been reported [43]. In recent years, preliminary progress has been made in the application of biological techniques such as *C. oleifera* somatic embryogenesis [44]. However, only a few *C. oleifera* cultivars induced callus suitable for protoplast isolation, which limited the application of callus and suspension cell lines protoplast isolation system to other *C. oleifera* cultivars. Compared with callus and suspension cell lines, leaves of in vitro grown seedlings are easier to obtain and widely used for plant protoplast isolation [18]. Any efficient transient expression system using mesophyll protoplast in *C. oleifera* had not been reported yet.

In this study, a highly repeatable and efficient protocol for mesophyll protoplast isolation and PEG-mediated transient transformation system was developed using *C. oleifera* leaves as starting materials. This protocol will provide a facile tool for protein subcellular localization and bimolecular fluorescence complementation assays as well as other in vivo molecular studies.

## Materials and methods

### Plant material and growth conditions

**Fig. 1:**
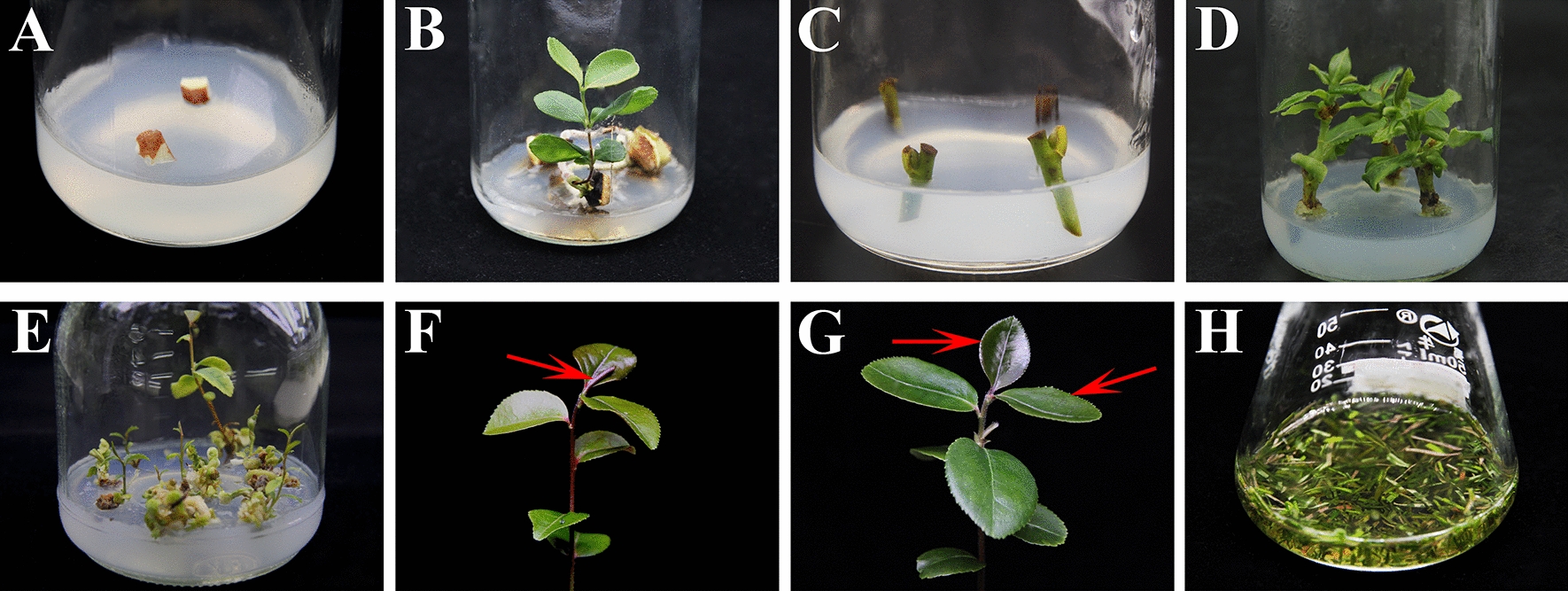
Leaf selection and treatment of in vitro growth seedlings of *C. oleifera*. **A** Seed primary generation culture; **B** seeds sprout into seedlings; **C** primary culture of stem segments; **D** axillary bud germination of stem segment; **E** proliferation culture of tissue culture seedlings; **F** undeveloped leaf; **G** the 1st to 2nd true leaf; **H** *C. oleifera* leaves sliced into 0.5 – 1.0 mm strips with a fresh razor blade and placed in EME media

*C. oleifera* ‘Huashuo’(HS), *C. oleifera* ‘TXP14’ and *C. oleifera* ‘DP47’ plant cultivars were obtained from the experimental base of Central South University of Forestry and Technology. In this study, the bud stems and seed embryos (Fig. 1A and C) for the three cultivars were used for culturing in vitro grown plantlets in MS (Murashige and Skoog) [45] medium for 40 days. When the bud stems and seed embryos were embryonic (Fig. 1B and D), they were kept on WPM (Woody Plant medium) [46] (pH 5.8) (Fig. 1E) containing 3.0% sucrose and 0.8% agar. Plants were kept at 25 ± 1 ℃, under a photocycle of 16 h/8 h (light (30 µmol·m^− 2^ ·s ^− 1^) /dark) for 6–8 week to obtain fully expanded leaves (Fig. 1F–G). First, the protocol for isolating the mesophyll protoplasts of *C. oleifera* was explored through ‘HS’ cultivar, and then the protocol was applied to ‘TXP14’ and ‘DP47’cultivars to verify the general applicability for protoplasts isolation in different cultivars of *C. oleifera*.

### Protoplast isolation

Young leaves of *C. oleifera *in vitro grown seedlings (subcultured for 1–2 years on the medium) were used to isolate protoplasts at room temperature. The in vitro grown seedlings were treated with dark. Then leaves of in vitro grown seedlings of different leaf ages were collected on the ultra-clean workbench and transferred into a sterile culture flask containing EME solution consisting of MS, 0.5% ME (malt extract), and different concentrations (0.3, 0.4, 0.5 and 0.6 M) of sucrose. The main veins and leaf margins were cut off with a sterile sharp blade, and then the leaves were cut into 0.5–1.0 mm wide strips (Fig. 1H). The strips were immediately transferred into 10 ml sterile EME medium solutions. After all the leaves were cut, the bottle sealing film was capped and placed in a vacuum pump for vacuum pretreatment under negative pressure (− 0.07 MPa). Then 5 ml of EME was pipetted out and 5 ml of enzyme solution was added to form a 10 ml enzymatic hydrolysis system. Enzyme solutions consist of 5.63 mmol/l MES, 24.49 mmol/l CaCl_2_·2H_2_O, 7.05 mmol/l NaH_2_PO_4_·2H_2_O, different concentrations (0.3, 0.4, 0.5 and 0.6 M) of mannitol, Cellulase R-10 (Yakult, Japan), Macerozyme R-10 (Yakult, Japan), Pectolyase Y-23 (Shanghai yuan ye Bio-Technology Co., Ltd, China), Hemicellulase (Shanghai regal Biology Technology Co, Ltd, China) and Snailase (Shanghai regal Biology Technology Co, Ltd, China) as shown in Table 1. All enzyme solutions were adjusted to pH 5.8, filter-sterilized through a 0.22 μm syringe filter (Millex-GP, USA), and then stored at 4 ℃. The digestion was performed at 28 ℃ by gently shaking (40 rpm) in the dark. The key parameters affecting protoplast isolation were tested, including osmotic pressures (0.3, 0.4, 0.5, 0.6 M mannitol), dark pretreatment time of in vitro grown seedlings (0, 12, 24, 30, 36, 40 h), vacuum pretreatment time (0, 10, 20, 30, 60 min), leaf age (unexpanded leaves, the 1st to 2nd true leaves and the 3rd to 4th true leaves), and enzyme digestion time (2, 4, 6, 8, 10, 12, 14, 16 h).

### Purification of protoplasts

After enzymatic digestion, the protoplasts were purified at room temperature by a combination of filtration, centrifugation and washing. The crude protoplast suspension was filtered through 200 mesh sterile steel sieve to exclude undigested tissues, cell clumps and cell wall debris. The filtrate was collected in a sterile centrifuge tube, and protoplasts were collected at low speed or natural rest. The mesophyll protoplasts of *C. oleifera* were purified by interfacial method and centrifugal precipitation. The interface method was similar to purifying protoplasts from *C. oleifera* suspension cells.

The protoplasts were resuspended in approximately 1:3 volumes with CPW14 salt solution (CPW14 salt solution contains 0.2 mmol/l KH_2_PO_4_, 1 mmol/l KNO_3_, 2.08 mmol/l MgSO_4_, 0.001205 mmol/l KI, 0.000012 mmol/l CuSO_4_·5H_2_O, 1.35 mmol/l CaCl_2_ and 400 mmol/l sucrose), CPW Ficoll 70 (Ficoll 70, Shanghai yuan ye Bio-Technology Co., Ltd, China) salt solution and CPW Ficoll 400 (Ficoll 400, Shanghai yuan ye Bio-Technology Co., Ltd, China) saline solution, respectively. Then CPW7 salt solution (CPW7 salt solution contains 0.2 mmol/l KH_2_PO_4_, 1 mmol/l KNO_3_, 2.08 mmol/l MgSO_4_, 0.001205 mmol/l KI, 0.000012 mmol/l CuSO_4_·5H_2_O, 1.35 mmol/l CaCl_2_ and 400 mmol/l mannitol) was gently added on top of it and centrifuged at 15 × g for 3 min to observe the purification effect.

Centrifuge precipitation was to gently add 4 ml W buffer (2 mmol/l MES, 125 mmol/l CaCl_2_, 5 mmol/l KCl, 154 mmol/l NaCl, 5 mmol/l glucose, pH 5.8) to the collected protoplasts, centrifuged at 15 ×*g* for 4 min, and then the supernatant was discarded. The pellet was resuspended with 4 ml of W buffer, and then the filtrate was centrifuged for 3 min at 50 ×*g*. After washing twice with the W buffer, the collected protoplasts were resuspended in 2 ml MMg solution (4 mmol/l MES, 0.4 mol/l mannitol, 15 mmol/l MgCl_2,_ pH 5.8), incubated on ice for 15 min.

### Protoplast yield and viability assessment

Purified protoplasts were counted using a blood cell count chamber under Olympus CX21 light microscope (Olympus, Japan). The yield was expressed as the number of protoplasts per gram fresh weight (g· FW). The viability was determined by fluorescein diacetate (FDA, Sigma-Aldrich, St. Louis, USA) staining according to Widholm [47]. The samples were incubated in dark for 5 min and then assessed under DMi8 inverted microscopy (Leica, Germany) with UV excitation light. Only viable protoplasts fluoresced bright green. The viability of the protoplasts was calculated by (viable protoplasts/total number of protoplasts) × 100%. For each sample, 3000 cells were analyzed in each replicate, and the counting was performed at least three times.

### Protoplast transformation

The pCAMBIA1300-GFP vector (supplementary information, additional file [1]) was used to test the transformation efficiency of the *C. oleifera* mesophyll protoplasts. For each assay, different amounts of plasmid DNA (5, 10, 15, and 20 µg) were added to 100 µl prepared protoplast (about 2 × 10^6^/g·FW protoplasts) and mixed gently. An equal volume of freshly prepared PEG solution (PEG, 0.3 M mannitol and 0.2 mol/l CaCl_2_) was immediately mixed with the protoplasts by shaking gently. PEG with different molecular weights (PEG3350, PEG4000, PEG6000, Sigma) and different final concentrations (20%, 30%, 40% and 50%) were tested. To optimize transfection duration, the mixture was incubated at room temperature for 10, 15, 20, and 25 min in the dark, respectively. After incubation, the transfection process was stopped by adding 200 µl W5 solution at room temperature. The mixture was centrifuged at 50 ×*g* for 1 min and the protoplasts were gently resuspended with 100 µl WI solution (4 mM MES, 0.4 M mannitol, 20 mmol/l KCl, pH 5.8). The transfected protoplasts were incubated at 25 °C in the dark for 12–16 h. The protoplasts expressing GFP-fusion were observed and images were captured using a confocal laser scanning microscope (Leica TCS SP8, Germany). The GFP fluorescence signals were acquired using 488 nm excitation wavelengths and 507 nm emission wavelengths. The exploration of each condition in GFP transformation experiment was performed at least three independent replicates. Transformation efficiency was calculated as follows: transformation efficiency (%) = (the number of bright green fluorescent protoplast in view/total number of protoplasts in view) × 100%.

### Statistical analysis

All data were performed with SPSS Version 18.0 (SPSS Inc. Chicago, IL, USA). One-way analysis of variance (one-way ANOVA) with a post hoc test of least significant difference (LSD) test was used for the statistical analysis. Data were presented as the mean value ± standard error (SE) from three independent experiments. *P* < 0.05 was considered to indicate a statistically significant difference.

## Results

### Effect of osmotic pressure on mesophyll protoplasts isolation in ***C. oleifera***

**Fig. 2:**
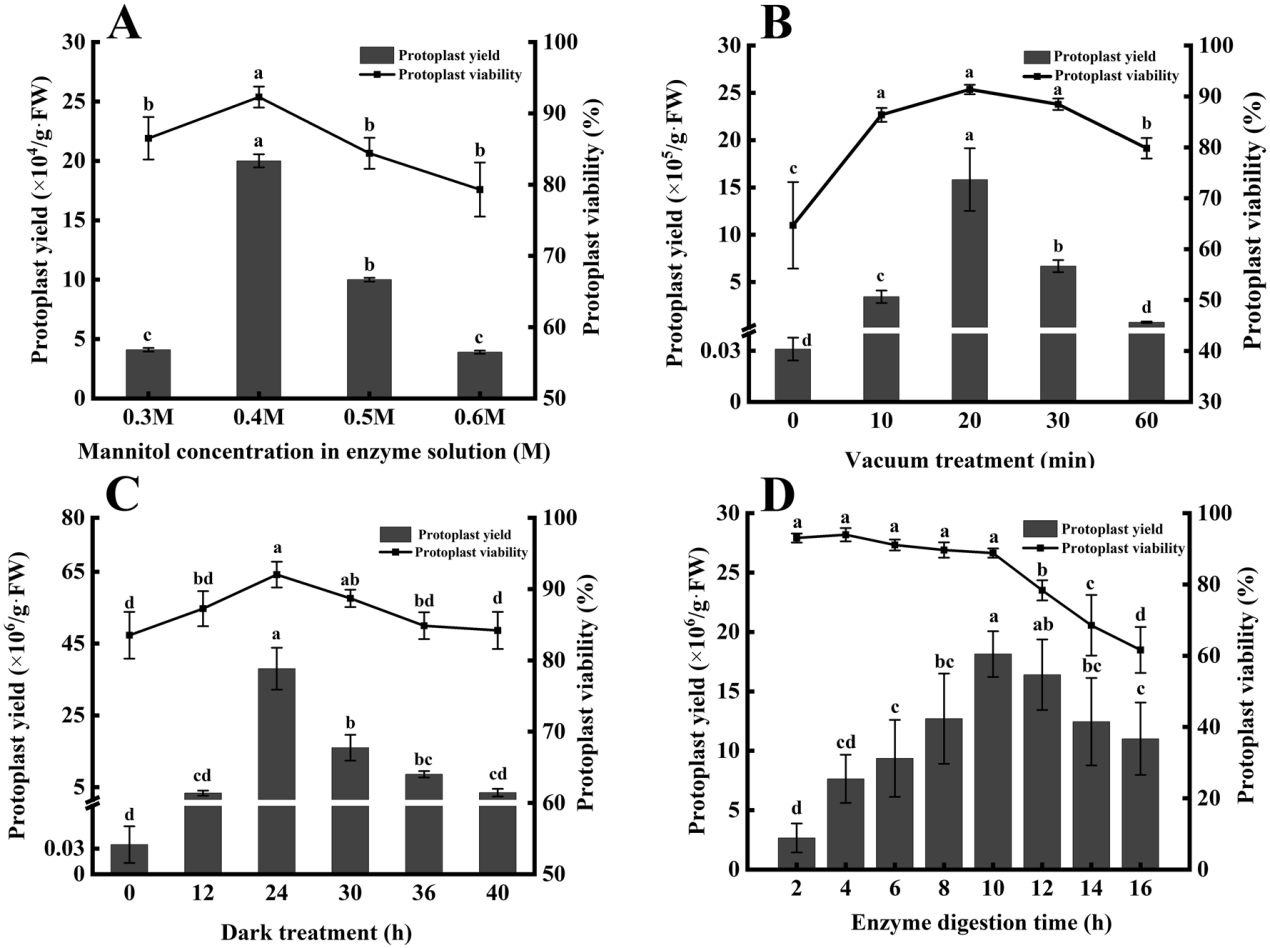
Effects of mannitol concentration, pretreatment method and duration of enzyme application on mesophyll protoplasts isolation in *C. oleifera*. **A** Effects of mannitol concentration in enzyme solution (enzyme composition in 1.0% Cellulase R-10 and 1.0% Macerozyme R-10) on protoplast isolation; **B** effects of vacuum treatment on mesophyll protoplasts isolation in *C. oleifera* (Vacuum treatment 1 –5 indicates that the vacuum treatment time is 0, 10, 20, 30 and 60 min respectively); **C** effects of dark treatment on mesophyll protoplasts isolation in *C. oleifera* (Dark treatment 1–6 indicates that the dark treatment time is 0, 12, 24, 30, 36 and 40 h, respectively); **D** effects of duration in enzyme application (enzyme composition in 1.5% Cellulase R-10, 0.5% Macerozyme R-10 and 0.25% Snailase) on protoplast isolation. Different letters represent a statistically significant difference at *P* < 0.05, and bars represent standard errors

The effects of different osmotic pressures on protoplast isolation of *C. oleifera* mesophyll were investigated using mannitol as an osmotic pressure regulator. The results showed that the isolation effect of *C. oleifera* mesophyll protoplasts increased initially and then decreased with the increase of osmotic pressure. Under the condition of low osmotic pressure (0.3 M mannitol), the protoplast yield and viability were low. When the osmotic pressure was 0.4 M and the enzyme concentration was 1.0% Cellulase R-10 and 1.0% Macerozyme R-10 for 14 h, the protoplast yield and viability reached the highest value, which was 2.0 × 10^5^/g·FW and 90.3%, respectively. When the osmotic pressure reached 0.5 M and 0.6 M (Fig. 2A), we observed deformed cells and increased cell debris, as well as decreased yield and declined viability. Therefore, we concluded the optimal osmotic pressure for the protoplast isolation of *C. oleifera* mesophyll was 0.4 M.

### Effect of pretreatment method on mesophyll protoplasts isolation in *C. oleifera*

The pretreatment methods are extremely important for the efficient release of protoplasts from *C. oleifera* leaves. It was found that the yield and viability of *C. oleifera* mesophyll protoplasts were affected by vacuum and dark pretreatment.

First, vacuum pressure was applied to enhance the infiltration of the enzyme digestion solution into the leaf blades. The leaves of *C. oleifera* were pretreated with a vacuum (− 0.07 MPa) for different time lengths. The results showed that the yield and viability of protoplasts were increased after vacuum treatment compared with those without vacuum treatment (Fig. 2B), and the protoplasts were complete in morphology, with more inclusions and fewer impurities. The results showed that vacuum pretreatment effectively promoted the enzymatic hydrolysis of *C. oleifera* leaves and improved the isolation efficiency of mesophyll protoplasts. Within a certain range of negative pressure, the mesophyll protoplast yield and viability of *C. oleifera* increased first and then decreased with the extended time of vacuuming. When the vacuuming treatment lasted for 20 min, the protoplast yield of *C. oleifera* mesophyll cells reached 1.5 × 10^6^/g·FW and the viability was 81.7%. Therefore, − 0.07 MPa vacuum pretreatment for 20 min is most suitable for the isolation of protoplasts from *C. oleifera* mesophyll.

In addition, protoplast yield and activity increased first and then decreased with the extended dark treatment (Fig. 2C). The protoplast yield reached 3.8 × 10^7^/g·FW and the protoplast viability reached 90.6% when the in vitro grown seedlings were treated in dark for 24 h. After dark treatment for more than 24 h, protoplast yield and viability began to decrease. Therefore, 24 h dark treatment is optimal for the isolation of *C. oleifera* mesophyll protoplasts.

### Effect of leaf age on mesophyll protoplasts isolation in *C. oleifera*

**Fig. 3:**
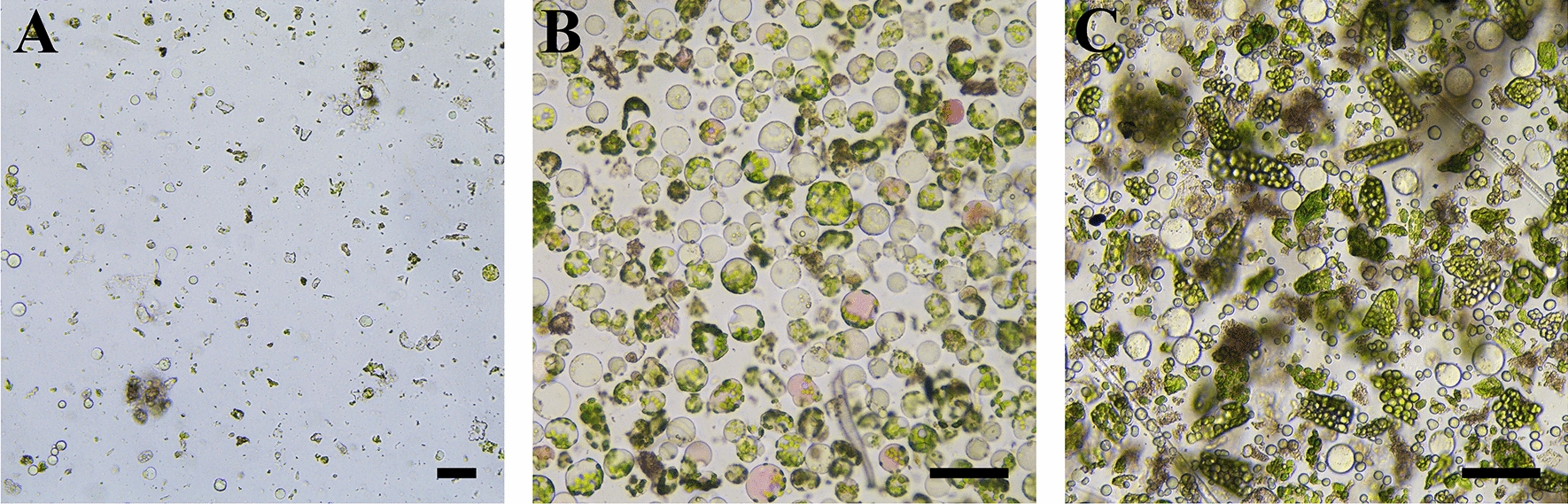
Efficiency of mesophyll protoplast isolation from leaves of different age collected from in vitro grown seedlings of *C. oleifera*. **A** Undeveloped leaves; **B** the 1st to 2nd true leaves; **C** the 3rd to 4th true 1eaves. The scale bars = 50 μm

The effect of leaf age on protoplast yield and viability was investigated. The leaves at different stages of *C. oleifera* growth (undeveloped leaves, the 1st to 2nd true leaves and the 3rd to 4th true leaves) were used. The results indicated that the age of the leaf tissue greatly affected the protoplast releasing. The protoplasts isolated from in vitro grown seedlings without undeveloped leaves (Fig. 1F) had a very low yield and were relatively easy to be broken (Fig. 3A). When the 1st to 2nd true leaves (Fig. 1G) were used, the yield of isolated protoplasts could reach 8.1 × 10^6^/g·FW (Fig. 3B), and the viability could reach 89.7% with other factors at optimal. However, the yield of protoplasts isolated from the 3rd to 4th true leaves was also low, accompanied by large amounts of debris and other irregular impurities (Fig. 3C). Compared with the 1st to 2nd true leaves, the yield and viability of protoplasts isolated from the 3rd to 4th true leaves were significantly reduced. Therefore, the 1st to 2nd true leaves of in vitro grown seedlings of *C. oleifera* should be the most appropriate for protoplast isolation.

### Effect of enzyme types, concentrations and digestion time on mesophyll protoplasts isolation in *C. oleifera*

The concentration and type of enzyme are critical for protoplast isolation. This study explored the effects of 10 enzyme combinations on the isolation of *C. oleifera* mesophyll protoplast (Table 1). In this study, the effects of 10 enzyme combinations on the isolation of *C. oleifera* mesophyll protoplasts (Table 1) were investigated when the osmotic pressure was 0.4 M and the enzyme digestion time was 14 h. We obtained the lowest yield of protoplasts when using 1.0% Cellulase R-10 and 1.0% Macerozyme R-10. In addition to the combination of Cellulase R-10 and Macerozyme R-10, a certain concentration of pectinase was added, then the yield of protoplasts was slightly increased. When the enzyme combination was 1.5% Cellulase R-10, 0.5% Macerozyme R-10 and 0.5% Hemicellulase, the protoplast yield was still low, along with the increase in cell debris. After many attempts, Hemicellulase was found not suitable for *C. oleifera* mesophyll protoplast isolation. Under the combination of Cellulase R-10, Macerozyme R-10 and Snailase, the yield of protoplasts was greatly increased. Further exploration of the optimal enzyme concentration of Cellulase R-10, Macerozyme R-10 and Snailase showed that treatment 7 had the best effect. The protoplast yield reached 3.5 × 10^7^/g·FW, and viability reached 90.9%. Therefore, the optimal combination of enzyme concentration for the *C. oleifera* mesophyll protoplast isolation was 1.5% Cellulase R-10, 0.5% Macerozyme R-10 and 0.25% Snailase.

**Table 1:** Effect of different enzyme concentration combinations on mesophyll protoplast isolation in *C. oleifera*

Treatment No.	Enzyme solution combination(%)					Protoplast yield( × 10^6^/g·FW)	Protoplast viability(%)
	Cellulase R-10	Macerozyme R-10	Pectolyase Y-23	Hemicellulase	Snailase		
1	1.0	1.0	0	0	0	0.07 ± 0.003d	61.7 ± 5.32d
2	1.0	1.0	0.25	0	0	0.61 ± 0.064 cd	83.1 ± 1.40abc
3	1.5	0.5	0.25	0	0	0.78 ± 0.045 cd	76.6 ± 1.38c
4	1.5	1.0	0.05	0	0	0.77 ± 0.048 cd	81.5 ± 3.35abc
5	1.5	0.5	0	0.5	0	0.08 ± 0.003d	62.4 ± 5.04d
6	1.5	0.5	0	0	0.2	20 ± 4.92b	89.65 ± 1.27a
7	1.5	0.5	0	0	0.25	35 ± 4.05a	90.9 ± 2.18a
8	1.0	1.0	0	0	0.25	19 ± 3.15b	87.2 ± 1.43ab
9	1.5	1.25	0	0	0.2	7.7 ± 0.512c	80.1 ± 1.16abc
10	1.5	1.25	0	0	0.25	6.2 ± 0.698 cd	79.7 ± 1.33bc

Different letters represent a statistically significant difference at *P <* 0.05, and bars represent standard errors

To establish the optimal time for enzyme treatment, we digested leaves for 2–16 h. The results indicated enzyme digestion time has a significant influence on both the yield and viability of protoplasts isolated from leaves of *C. oleifera*. As enzyme digestion time increased from 2 to 10 h, protoplast yield increased gradually, peaked at 10 h and decreased significantly with further extension in enzyme digestion time (Fig. 2D). Based on these results we concluded that the optimal enzyme digestion time was 10 h for isolating *C. oleifera* mesophyll protoplasts.

### Effects of purification method and CPW solution on protoplast purification

**Fig. 4:**
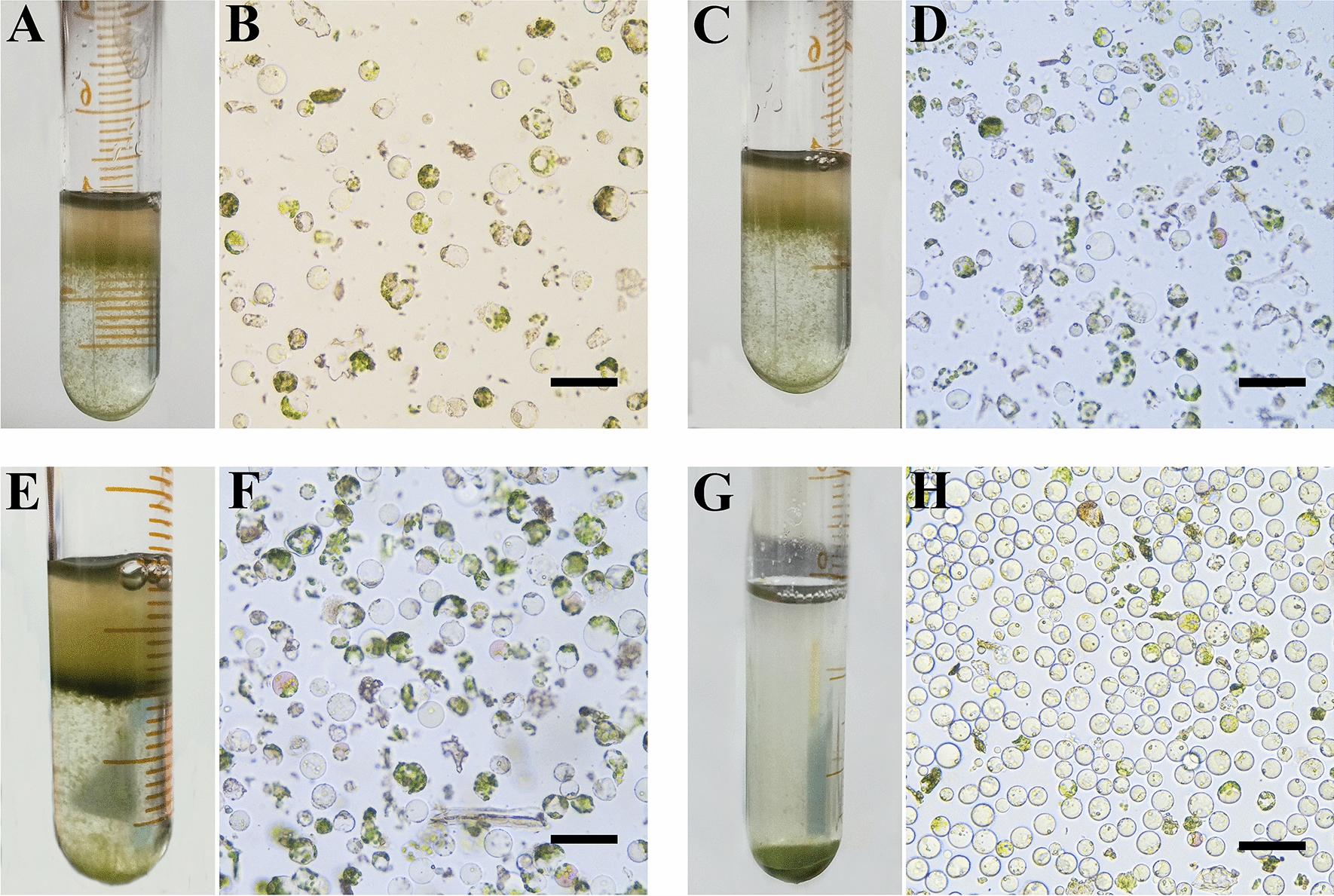
Purification of *C. oleifera* mesophyll protoplasts. **A**, **B** CPW14 purification; **C**, **D** CPW Ficoll 400 purification; **E**, **F** CPW Ficoll 70 purification; **G**, **H** W buffer purification; **A**–**F**: the interface purification method; **G**–**H**: precipitation method. The scale bars = 50 μm

**Fig. 5:**
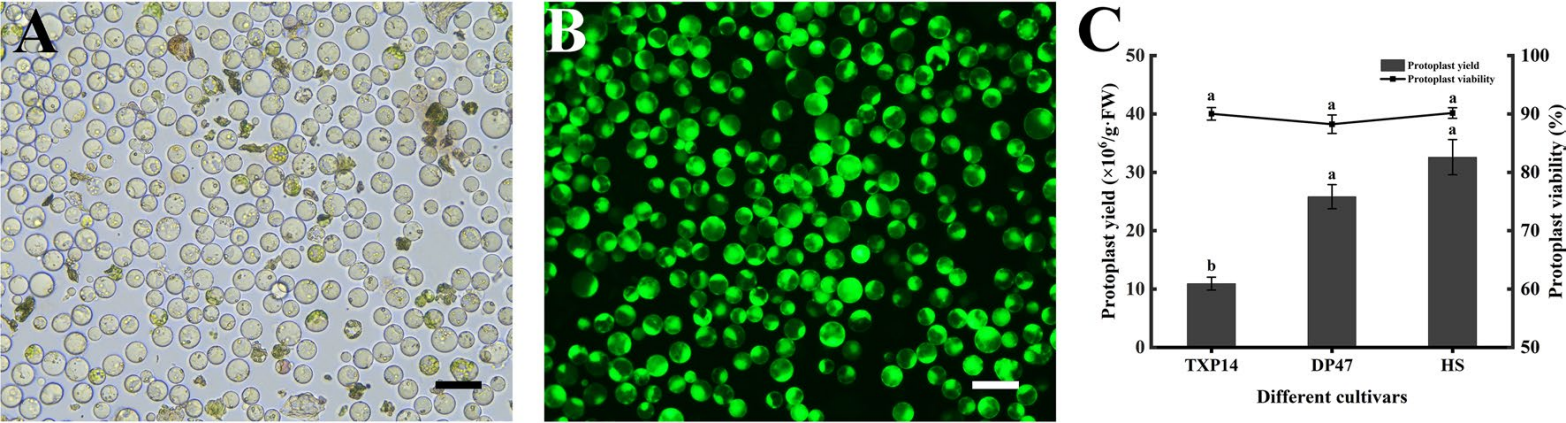
Determination of mesophyll protoplast viability in *C. oleifera* and the effects of different cultivars on mesophyll protoplast isolation of *C. oleifera*. **A** FDA dyed protoplast under the bright light; **B** FDA dyed protoplast under the ultraviolet light. **C** The effects of different cultivars on mesophyll protoplast isolation of *C. oleifera*. The scale bars = 50 μm

On the basis of protoplast purification from the cell suspension, the purification method of mesophyll protoplast of *C. oleifera* was explored. Firstly, the mesophyll protoplasts were purified by the interface method. CPW14, CPW Ficoll 70 and CPW Ficoll 400 buffer solutions were used to resuspend the mesophyll protoplasts, and then the CPW7 was added gently in a ratio of 3:1 to form stratification between the two liquids. After centrifugation, protoplasts are expected to accumulate at the interface in the form of clear strips (Fig. 4). Although the purification effect of CPW Ficoll 400 was better than that of CPW Ficoll 70 and CPW14, none of the three solutions was ideal due to the presence of a large number of cell debris and other impurities. The mesophyll protoplasts were further purified by centrifugal precipitation using W buffer as a cleaning agent, and relatively pure and highly active mesophyll protoplasts were obtained (Fig. 4H). The results showed that using W buffer as a cleaning agent by centrifugal precipitation was optimal, and the viability of purified protoplasts was as high as 90.9% (Fig. 5A, B).

### Effects of different *C. oleifera* cultivars on mesophyll protoplasts isolation

To verify the applicability of the present protocol, protoplasts were isolated from the 1st to 2nd true leaves of the other two *C. oleifera* cultivars (‘TXP14’ and ‘DP47’). The protoplast yield for ‘TXP14’ and ‘DP47’ was 1.1 × 10^7^/g·FW and 2.6 × 10^7^/g·FW, the protoplast viability for ‘TXP14’ and ‘DP47’ was 90.0% and 88.2%. Therefore, an effective protocol for isolating and purifying protoplasts from *C. oleifera* plants was established, and the effect of protoplast isolation in different *C. oleifera* cultivars was verified.

### Transient transformation efficiency in *C. oleifera* mesophyll protoplasts

**Fig. 6:**
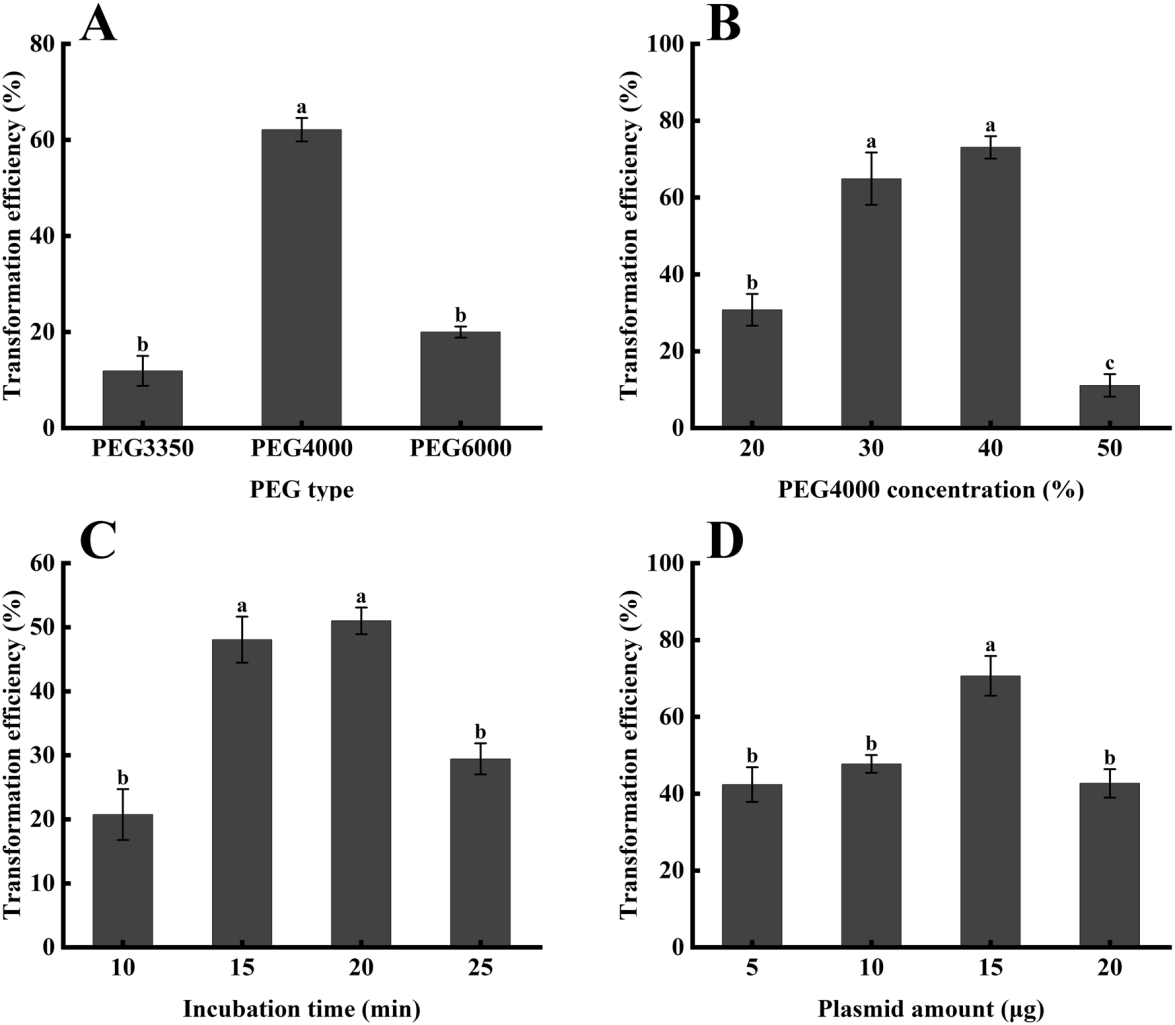
Efficient transfection of *C. oleifera* mesophyll protoplasts. Effects of PEG molecular weights (**A**), PEG4000 concentration (**B**), incubation time when plasmid amount was 10 µg (**C**), and plasmid amount (**D**) on *C. oleifera* protoplast transformation efficiency. Different letters represent a statistically significant difference at P < 0.05, and bars represent standard errors

The effects of PEG4000 concentration and plasmid amount on transformation efficiency of *C. oleifera* mesophyll protoplasts were assessed using the pCAMBIA1300-GFP vector. To optimize PEG molecular weights, the effect of PEG molecular weights (PEG3350, PEG4000, PEG6000) on transformation efficiency was examined when the PEG concentration was 30%. The transformation efficiency was approximately 18% at PEG3350 (Fig. 6A), and the transformation efficiency improved with increasing PEG molecular weights. The transformation efficiency reached 58.2% at PEG4000. Then, as the PEG molecular weights continued to increase, the transformation efficiency dropped sharply. Thus, PEG4000 was regarded as the optimal PEG molecular weights for transient expression using *C. oleifera* mesophyll protoplasts. As shown in Fig. 6B, transformation efficiency first increased, then declined, along with increased PEG4000 concentration (20%, 30%, 40% and 50%, respectively). When PEG4000 was at concentration of 40%, transformation efficiency reached the maximum, approximately 73.07%. Subsequently, transformation efficiency reduced gradually. When PEG4000 concentration was 50% the transformation efficiency decreased to 11.11%, and the ratio of abnormal protoplasts rose and protoplast debris increased. In conclusion, 40% was the optimal concentration of PEG4000.

The effects of PEG incubation time (10, 15, 20, and 25 min) on the transformation efficiency were analyzed (Fig. 6C). Increasing the transfection time from 10 to 20 min led to an increase in the transformation efficiency from 20.7 to 51.0%. However, the continued prolongation of transfection decreased the efficiency, indicating that the optimal incubation time for protoplast transient transformation was 20 min. To investigate the effect of plasmid amount on the transformation efficiency of *C. oleifera* mesophyll protoplasts, 5, 10, 15, and 20 µg of pCAMBIA1300-GFP vector were tested in 100 µl resuspended protoplasts in WI. The results showed that when plasmid concentration was 5 µg, the transformation efficiency was 42.37% (Fig. 6D). As plasmid amount increased, transformation efficiency increased as well and reached 70.66% at 15 µg. However, when plasmid amount was further increased to 20 µg, transformation efficiency decreased significantly to 42.7%. This indicated that the optimal plasmid amount for transient transformation was 15 µg.

**Fig. 7:**
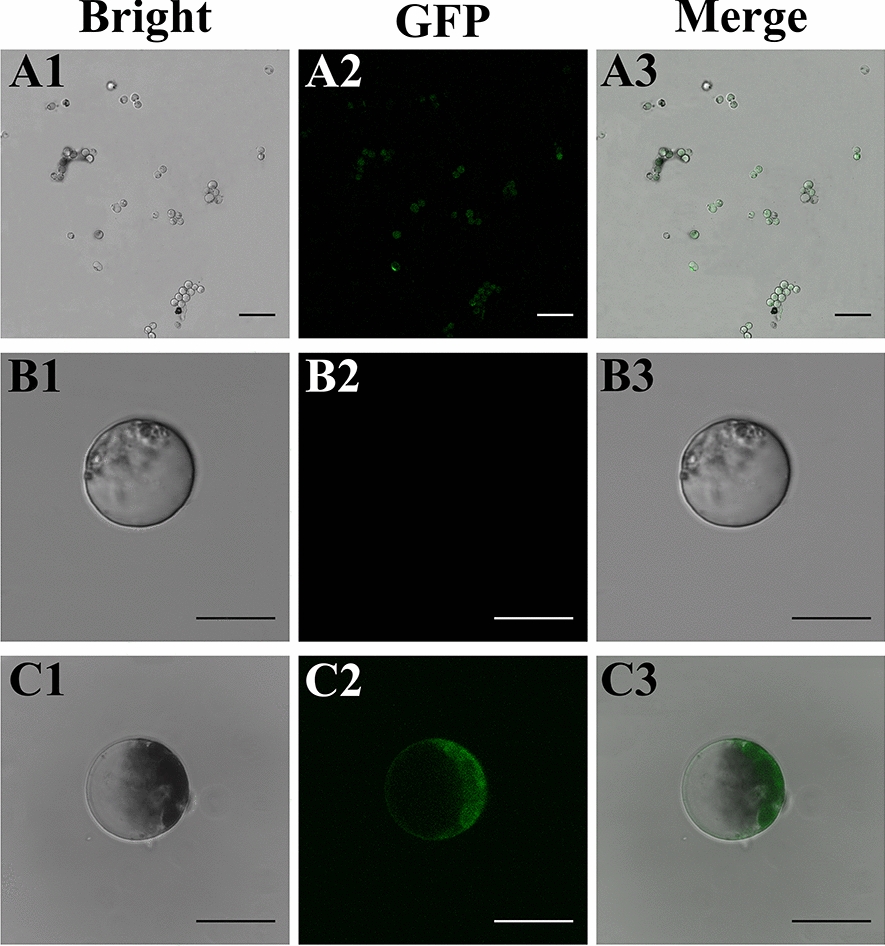
Transient expression of GFP in *C. oleifera* protoplasts. **A1**–**A3** Efficiency transformation of *C. oleifera* mesophyll protoplasts with GFP plasmid (The scale bars = 100 μm); **B1**–**B3** Unsuccessful transient expression of GFP in *C. oleifera* mesophyll protoplasts. **C1**–**C3** Successful transient expression of GFP in *C. oleifera* mesophyll protoplasts. Bright: bright field image of protoplasts; *GFP* green fluorescent protein; Merged: GFP merged with chloroplast autofluorescence (The scale bars = 20 μm)

Based on the obtained data, the optimal protocol of transformation in *C. oleifera* protoplast was found to be incubated with 40% PEG4000 and 15 µg plasmid for 20 min of transfection time. Using this method, a maximum transformation efficiency of approximately 70.6% (Fig. 7A1–A3) was obtained from *C. oleifera* mesophyll protoplasts. In addition, it was further found that the protoplasts transformed by the GFP vector under bright field were regular in shape, and the cell membrane was intact. At the excitation light of 488 nm, no auto-fluorescence signal could be observed in the untransformed protoplasts (Fig. 7B1–B3). The GFP-expressing region in the transformed protoplast showed obvious green fluorescence, indicating that the plasmid containing the *gfp* gene could be introduced into the *C. oleifera* mesophyll protoplasts and expressed transiently (Fig. 7C1–C3).

## Discussion

*C. oleifera* is a widely distributed plant species in southern China [1], with a planting area of more than 4.5 million hectares [48]. The *C. oleifera* industry is rapidly expanding and developing, becoming one of the main industries for rural revitalization in China [4]. However, the lack of improved varieties and backward breeding technology limit the development of *C. oleifera* industry. Somatic hybridization is one of the promising technologies in advancing *C. oleifera* breeding. The protoplast system is the basis of somatic hybridization, and is also an important scientific research tool, which provides the possibility for molecular assisted breeding and molecular design breeding of *C. oleifera*. In this study, an optimal system for the isolation and purification of mesophyll protoplasts from *C. oleifera *in vitro grown seedlings was established.

Mesophyll tissues of leaves are one of the most convenient sources for a large number of uniform cells for protoplast isolation [18, 28]. The yield of protoplasts was influenced by the physiological state and growth cycle of plant leaves. In woody plants, young tissues have consistently proved to be the best sources for protoplast isolation [49]. Protoplast yield drops sharply when isolated from old leaf tissue [50]. Thus, the age of tissue plays a critical role in the yield and viability of protoplasts. Furthermore, the suitable leaf age for mesophyll protoplast isolation was different among different plants. For example, the optimal leaf for wheat protoplast isolation is 7 days old [6], while for *Arabidopsis* is 3–4-week [18], and for cotton is 12 days [51]. Moreover, it has shown that the enzyme digestion time of unexpanded leaves was not easy to control, and also produced a large amount of cell debris [6], while the viability of protoplasts obtained in older leaves was lower [17]. Therefore, in this study, the 1st to 2nd true leaves of the in vitro grown seedlings of *C. oleifera* were the most suitable for the mesophyll protoplasts isolation.

Pretreatment of source tissue before enzymatic hydrolysis could change the physiological state of cells and cell walls and reduce the loss of protoplasm [52]. In the process of mesophyll protoplast isolation, pretreatment methods such as vacuuming, pre-plasmolysis, dark and low-temperature pretreatment were often used. Choury et al. found that vacuuming the leaves of *Arbutus unedo* for 30 min [53], and Rahmani et al. [54] treated *Albizia julibrissin* leaves or callus in 0.7 M sorbitol for 60 or 90 min could improve the isolation efficiency of the protoplast. Furthermore, Chang et al. [55] found that dark pretreatment was necessary for successful protoplast isolation from potato leaves. Liao et al. [56] found that at 4 °C low-temperature pretreatment could increase the viability of *Arabidopsis* mesophyll protoplasts. Previous studies have shown that vacuuming pretreatment was the most commonly used for the isolation of plant mesophyll protoplasts, such as sugarcane [57], *Phaseolus vulgaris* [37], and *Brachypodium distachyon* [58]. The pretreatment methods for the isolation of *C. oleifera* and tea (*C. sinensis*) protoplasts were not identical in *Camellia* genus. Xu et al. [59] reported that the efficiency of protoplast isolation in tea was improved by vacuuming treatment. Peng et al. [24] successfully obtained mesophyll protoplasts from tea seedlings grown in the dark. Previously, we have found that no pretreatment was required when isolating protoplasts from *C. oleifera* suspension cells [43]. In this study, the optimal pretreatment of *C. oleifera* leaves was dark treatment for 24 h and negative 0.07 MPa vacuum treatment for 20 min. It could be further speculated that different starting materials of the same genus and the same species of plants may need different pretreatments for protoplast isolation.

The concentration of osmotic stabilizers required for successful protoplasts isolation varied among the plant species and growing conditions [60]. For example, in barley, 0.3 M mannitol was found to be optimal for the high yield and viability [41]. Previous studies have shown that the optimum mannitol concentrations for *Catalpa bungei*, sorghum and Chinese kale for protoplast isolation are 0.4 M [61], 0.5 M [62] and 0.6 M [63], respectively. Furthermore, it was found that the optimal osmotic pressures for the isolation of *Phalaenopsis aphrodite* and bamboo mesophyll protoplasts were 0.7 M [64] and 0.8 M [65], respectively. In addition, studies have found that the osmotic pressure of different tissues of the same species could be the same or different. For example, the optimal osmotic pressure for grape mesophyll protoplast isolation is 0.6 M, while the optimal osmotic pressure for callus tissue is 0.5 M [66]. Peng et al. [24] found that the optimal osmotic pressure for protoplast isolation between young leaves and young radicles of tea plants is 0.4 M. In the study, it was found that 0.4 M mannitol was most suitable for mesophyll protoplast isolation of *C. oleifera*, which was consistent with the previous studies of *C. oleifera* suspension [43] and *C. sinensis* plant [24, 59]. In conclusion, 0.4 M may be a suitable osmotic pressure for *Camellia* plants (Additional file 1).

It has reported that appropriate enzyme digestion time and enzyme combination are crucial for protoplast isolation [67]. The composition of the enzyme solution and the enzymatic hydrolysis time required for protoplast isolation from different plants were generally different. Zhou et al. [25] found that the most mesophyll protoplasts were obtained from tea digested with 3% cellulase R-10 and 0.3% macerozyme R-10 for 12 h. While the optimal conditions for mesophyll protoplast isolation of *Platycladus orientalis* were 1.5% cellulase R-10, 0.4% macerozyme R-10, 0.4% pectolyase Y-23 and 1.0% ligninase for 16 h [68]. However, compared with woody plants, the enzyme concentration and enzyme time required for protoplast isolation of herbaceous plants were lower. Li et al. [69] found the highest yield and viability *Phalaenopsis* protoplasts were achieved with 1.0% Cellulase Onozuka R-10, 0.7% Macerozyme R-10 for 6 h. Adedeji et al. [70] found that a high *Chrysanthemum* protoplast yield was achieved using 1.5% cellulase, and a 4 h incubation period. It further suggested that the isolation of woody plant protoplasts required higher enzyme concentration, longer enzyme time and even some special enzymes. It was well known that protoplast isolation technology was underdeveloped in woody plants compared with herbaceous plants [25]. These differences might be due to the differences in cell wall composition and biological activity of cells, resulting from differences in the physiological characteristics of plants and growth environments [30].

In the process of protoplast isolation, no matter how efficient the enzymatic hydrolysis system was, a lot of impurities such as cell debris would always be produced. These impurities would have a negative impact on protoplast culture and transformation. Therefore, protoplasts must be purified to remove impurities. There are three commonly used methods for protoplast purification, namely centrifugal precipitation method, floating method and interface method. Different plant protoplast purification methods were different, the protoplasts of cucumber [71] and *Catalpa bungee* [61] were purified by centrifugal precipitation. *Pisum* and *Lathyrus* protoplasts were purified by floating method [72]. Mango protoplasts [73] and sweet cherry protoplasts [67] were purified by the interface method. In our study, it was found that the purification methods of mesophyll protoplasts and suspension cells protoplasts of *C. oleifera* were different, which might be due to the differences in contents, cell density and the other states of protoplasts isolated from the two explants. Generally, protoplast purification operation would reduce protoplast yield and viability, which was very important for subsequent protoplast culture, regeneration and genetic transformation. The mesophyll protoplast activity of *C. oleifera* isolated and purified by the method of this study reached 90.9%, which lays a good foundation for subsequent research such as somatic hybridization and gene editing.

PEG-mediated transient transformation of plant protoplasts is widely used in plants, but the transfection efficiency varies greatly among different plant species [17, 40]. Firstly, the effect of PEG molecular weights on transformation efficiency was explored. The result showed that the transformation efficiency is higher when PEG4000 was used. As PEG4000 concentrations increased, the transformation efficiency rose significantly, but impurities such as cell debris increased as well, which may inhibit the transformation efficiency [71]. For example, the optimum PEG4000 concentration for *Populus* and cassava have been reported to be 30% and 25%, respectively [26, 34]. We found that 40% PEG4000 is optimal for the transformation of protoplasts derived from *C. oleifera*. In addition, the optimum amount of plasmid for protoplast transient transformation is different in different species [42, 74]. Different amounts of plasmids, such as 20 µg for *Brachypodium distachyon*, and 10 µg for soybean, have been reported to be the optimal amounts of plasmid DNA in their established protocols respectively [58, 75]. Our assay demonstrated an increased transformation efficiency could be obtained with an increase in plasmid amount in *C. oleifera*, but it reached a plateau at 15 µg. Thus, 15 µg was considered to be the optimal amount of plasmid for the present *C. oleifera* protoplast transformation. The optimal incubation time for different species is different, such as 5 min for grapevine [42], 10 min for cassava [34], 15 min for Chinese kale [63], 20 min for barley [41], and 30 min for cucumber [71] protoplasts. The effect of incubation time on transformation efficiency was also explored in this study. The highest transformation efficiency was obtained when the *C. oleifera* protoplasts were incubated for 20 min.

## Conclusion

In summary, a highly efficient protocol for *C. oleifera* mesophyll protoplast isolation and PEG-mediated transient expression was developed. To our knowledge, this is the first report describing the isolation of mesophyll protoplasts from the *C. oleifera* and of the PEG-mediated protoplast transfection. The developed method could be a convenient technique for protein subcellular localization, promoter function validation, and many other molecular biology studies in *C. oleifera*.

## Supplementary Information

**Additional file 1. **The schematic representation of the T-DNA region of pCAMBIA1300-GFP vector.

## Data Availability

The datasets supporting the conclusions of this article are included in the article.
