# Supplementary figures and images for: Isolation, purification and PEG-mediated transient expression of mesophyll protoplasts in Camellia oleifera
Source: Plant Methods. 2022 Dec 22;18:141. doi: 10.1186/s13007-022-00972-1 (PMC9773467; doi:10.1186/s13007-022-00972-1)

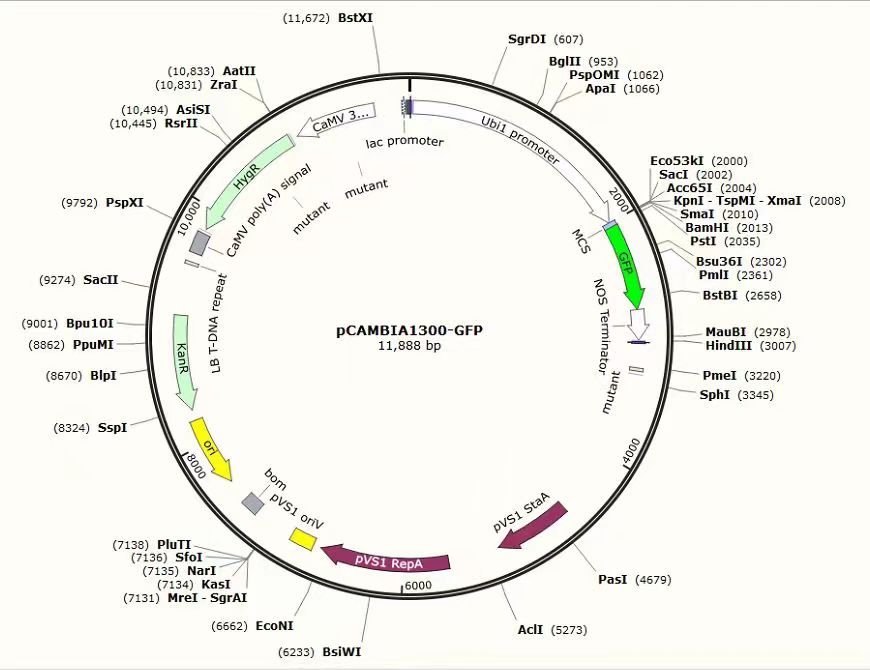


The schematic representation of the T-DNA region of pCAMBIA1300-GFP vector.

Supplement: Supplementary file 1 — Additional file 1. The schematic representation of the T-DNA region of pCAMBIA1300-GFP vector. [file 13007_2022_972_MOESM1_ESM.docx]
